# Sequential therapeutic targeting of ovarian Cancer harboring dysfunctional BRCA1

**DOI:** 10.1186/s12885-018-5250-4

**Published:** 2019-01-10

**Authors:** Tahira Baloch, Vanessa M. López-Ozuna, Qiong Wang, Emad Matanis, Roy Kessous, Liron Kogan, Amber Yasmeen, Walter H. Gotlieb

**Affiliations:** 1 Division of Gynecologic Oncology, Jewish General Hospital, McGill University, 3755 Cote Ste. Catherine Road, Montreal, QC H3T 1E2 Canada; 2 Segal Cancer Center, Lady Davis Institute of Medical Research, McGill University, Montreal, QC Canada; 3 Department of Experimental Surgery, McGill University, Montreal, QC Canada; 4 Department of Experimental Medicine, McGill University, Montreal, QC Canada

**Keywords:** Ovarian Cancer, BRCA1/2, PARP inhibitor, Chemoresistance, DNA repair pathway, Synthetic lethality

## Abstract

**Background:**

Poly (ADP-ribose) polymerase inhibitors (PARPi) have become the first targeted therapies available in the treatment of patients with high-grade serous ovarian cancer (HGSOC). We recently described a significant reduction in PARP1 protein levels in vitro and in vivo in patients treated with standard carboplatinum-paclitaxel chemotherapy, raising the question whether the sequence of treatment used today with chemotherapy followed by PARPi is optimal. In this study, we aim to evaluate if the sequence of PARPi followed by chemotherapy could be more beneficial.

**Methods:**

BRCA1-mutated (UWB1.287, SNU-251), epigenetically-silenced (OVCAR8), and wild-type (SKOV3, A2780PAR & A2780CR) ovarian cancer cell lines were exposed to clinically relevant doses of PARPi followed by different doses of standard chemotherapy and compared to the inverse treatment. The therapeutic efficacy was assessed using colony formation assays. Flow cytometry was used to evaluate cell apoptosis rate and the changes in cell cycle. Finally, apoptotic and cell cycle protein expression was immunodetected using western blot.

**Results:**

Exposure to PARPi prior to standard chemotherapy sensitized BRCA1-mutated or epigenetically-silenced BRCA1 cell lines to lower doses of chemotherapy. Similar results were observed in BRCA1 wild-type and cell lines in which BRCA1 functionality was restored. Moreover, this treatment increased the apoptotic rate in these cell lines.

**Conclusion:**

Pre-treatment with PARPi followed by standard chemotherapy in vitro is more efficient in growth inhibition and induction of apoptosis compared to the administration of standard chemotherapy followed by PARPi.

**Electronic supplementary material:**

The online version of this article (10.1186/s12885-018-5250-4) contains supplementary material, which is available to authorized users.

## Background

Ovarian cancer remains the leading cause of death from gynecological malignancies. This cancer is typically diagnosed in advanced stages and still represents a challenge due to poor overall survival [1]. Ovarian cancer (OC) is a heterogeneous disease that includes different histological subtypes with distinct clinicopathological features [2]. High-Grade Serous Ovarian Cancer (HGSOC) is the most common subtype (70%) [3] and the majority show a significant, but transient response to platinum-taxane therapy and debulking surgery. However most patients will relapse and develop resistance to treatment that will lead them to poor overall survival [4, 5].

A significant proportion of HGSOC contain mutations in genes involved in the homologous recombination pathway of DNA repair (HR), especially *BRCA1* and *BRCA2*. Both *BRCA1/2* mutation-associated tumors and tumors with HR deficiencies have higher response rates to platinum-based chemotherapy [6, 7]. The majority of HGSOC are initially sensitive to platinum-based chemotherapy, however up to 75% of responding patients will relapse and developed platinum-resistance disease resulting in poor 5-year survival [8, 9]. Upon disease relapse, patients will most often undergo multiple lines of chemotherapy regimens to control symptoms and improve survival. However, the response rates and disease-free intervals will decrease, ultimately developing drug resistance.

PARPi are presently approved as maintenance therapy following platinum and taxol chemotherapy [10, 11]. HGSOCs are ideal candidates for PARPi as they are highly enriched for BRCA mutations and HR deficiencies [12].

PARPi function by blocking PARP1 protein [13, 14] and inducing synthetic lethality in HR deficient cells [15–19]. Moreover, the use of PARPi in other cancers with HR repair deficiencies, such as breast cancer, pancreatic cancer, and prostate cancer are being explored as well [20, 21]. Olaparib was the first PARPi to be introduced as a maintenance treatment for ovarian cancer patients that harbor BRCA mutations [22]. Clinical activity was most commonly noted in the platinum-sensitive patient population, although individuals with platinum-resistant cancers were also documented to respond [23]. Furthermore, clinical activity has also been observed in HGSOC in the absence of a BRCA mutation, although the response rates are lower in this setting [24]. Other PARPi such as rucaparib [25] and niraparib [26] have been approved for clinical use [27].

The rationale of this study was triggered based on our previous findings that reported a dramatic reduction of the target of PARPi, the intratumoral PARP1 protein levels in HGSOC tumors obtained in patients after exposure to chemotherapy with platinum and paclitaxel [28]. It raised the question whether PARPi would be more effective if given before chemotherapy at a time where the PARP1 target is present at higher levels in the cells rather than after chemotherapy when the target is reduced. Here we demonstrate that giving PARPi before chemotherapy is more effective in vitro to inhibit growth and induce apoptosis, compared to giving chemotherapy followed by PARPi. Altogether highlight the use of PARPi in a different setting, which can improve patient outcome and decrease the chemoresistance.

## Methods

### Cells lines and treatments

SKOV3 (#HTB-77), UWB1.289 (#CRL-2945), SNU-251 (#CVCL-5040) and OVCAR8 (#CVCL-1629) were purchased from ATCC. UWB1.289-BRCA1 (#CRL-2946) was provided by Dr. Ramandeep (University of Detroit) [29]. A2780PAR (parental) and A2780CR (cisplatin resistant) cells were provided by Dr. Seftor (Northwestern University, Chicago, purchased from the European collection of cell cultures, ECACC via Sigma). All the cells lines were authenticated by short tandem repeat (STR) profiling by the DNA sequencing and analysis core of the University of Colorado which has extensive experience in evaluation of gynecological cell lines [30]. All cell lines were frequently tested for mycoplasma infection using MycoAlert Detection Kit (Lonza #LT07–710).

SKOV3, A2780PAR and A2780CR contain wild-type BRCA1 genes, while OVCAR8 contains methylated BRCA1 genes. UWB1.289-BRCA1 is homozygous for the 2594delC BRCA1 mutation and SNU-251 is homozygous for the 5564G > A BRCA1 mutation. SKOV3 and OVCAR8 cells were cultured in RPMI-1640 supplemented with 10% fetal bovine serum (FBS), 2 mM glutamine, and1% Penicillin/Streptomycin. UWB1.289 and UWB1.289-BRCA1 cells were cultured in 50% MEGM medium (supplemented with hEGF, BPE, insulin, hydrocortisone), 50% RPMI-1640 (supplemented with 10% FBS, 2 mM glutamine) and 1% Penicillin/Streptomycin. SNU-251 cells were cultured in DMEM supplemented with 10% FBS, and 1% Penicillin/Streptomycin. A2780PAR and A2780CR were cultured in RPMI-1640 medium supplemented with 10% FBS, 1% HEPES and 1% Penicillin/Streptomycin. Each cell line was passaged every 4 to 6 days depending on its growth. All cells were maintained at 37 °C in a 5% CO2, 95% atmosphere incubator. All assays were performed in their respective medium.

Olaparib (AZD2281 #A10111), rucaparib (AG014699 #A10045) and niraparib (MK4827 #A11026) were purchased from AdooQ Bioscience. The drugs were diluted in DMSO (10 mM stocks) and stored at − 20 °C. To avoid drug degradation, new aliquots were prepared directly from stocks every 5–10 uses. The concentration used in the present study ranged between 0.01–10 μM for all PARPi which are considered to be at the lower range of that used in the clinical trials based on blood plasma concentrations. Cisplatin was ordered from the Jewish General Hospital Satellite Pharmacy.

### Generation of stable cell lines

SKOV3, SNU-251 and OVCAR8 cells were used to generate stable cell lines for experiments with knockdown or restored BRCA1. Cells were seeded in 6 well flat bottom cell culture plates. Lipofectamine (Invitrogen, Burlington, Ontario, Canada) (1:1) was mixed with control shRNA, BRCAshRNA, pcDNA3 and pcDNA3-BRCA1 separately with their respective media with no FBS. Following 30 min of incubation at room temperature, both control and plasmids were added to their respective wells. The cells were incubated at 37 °C for 5 h. Pools of stable transfected cells were selected using 2 mg/ml puromycin for up to a week, as previously described [31].

### Protein extraction and western blot analysis

Cells were lysed using RIPA buffer (25 mM Tris.HCl, pH 7.6, 150 mM NaCl, 1% NP-40.0.25% sodium deoxycholate 0.1% SDS) supplemented with protease and phosphate inhibitor (PhosphoSTOP, Roche Diagnostics, Mannheim, Germany). Total Protein concentration was determined using a BCA assay kit (Ref 23,227, Pierce Thermo-Scientific, Rockford, IL, USA) and a spectrophotometer at 570 nm. Proteins were run on SDS-PAGE and transferred to nitrocellulose membrane for western blot analysis, using the appropriate antibodies. Primary antibodies: BRCA1 antibody (#9010 Cell Signaling), PARP1 (#9542 Cell Signaling), MDR1 (#12683 Cell Signaling), Bad Ser473 (#9271S Cell Signaling), Cleaved Caspase 3 (#9661 Cell Signaling), Bcl2 (#2876 Cell Signaling), p-Bad (#06–853 upstate Cell signaling), p-Bcl-2 (# 2875 Cell signaling) and β-actin (#4967 Cell Signaling). Secondary antibodies: anti-rabbit-HRP (L170–6515 Bio-Rad), anti-mouse HRP (L170–6516 Bio-Rad). Immunoblot proteins were visualized using horseradish peroxidase (HRP) conjugated secondary antibodies, and antigen-antibody complexes were detected using the ECL system.

### Survival assays

Cell survival was assessed by clonogenic assays. 800–1000 cells were seeded into 6-well plates and grown in culture media with Cisplatin and/or PARPi (olaparib, rucaparib and niraparib). DMSO was used as a control. After treatments cells were washed, trypsinized, counted and plated in 60 mm dishes in triplicate and incubated for 7–14 days. Colonies were stained with 6.0% glutaraldehyde and 0.5% crystal violet and counted. Each experiment was repeated five times. Cell colonies were counted by using a Gel Count (Oxford optronix, UK). The surviving fraction (SF) of cells was calculated as follows: number of colonies formed after treatment/number of cells seeded x plating efficacy. The plating efficacy was calculated as follows: number of colonies formed in control/number of cell seeded. Drug interaction was assessed using the multiple drug effects analysis method of Chou and Talalay [32]. This method quantitatively describes the interaction between two drugs or a combination of two drugs together, with values of less than 1 indicating synergistic interactions, value greater than 1 indicating antagonistic interactions, and the values equal to 1 additive interactions. Calculations of the combination index were performed with CompuSyn Software (ComboSyn, Inc., Paramus, NJ. 07652 USA).

### Annexin V/PI apoptosis detection assays

For annexin V/PI assay, cells were stained with annexin V and PI (propidium iodide), and evaluated for apoptosis and necrosis by flow cytometry, according to the manufacturer’s protocol (eBioscience™ Annexin V Apoptosis Detection Kit eFluor™ 450). Treated cells were collected using trypsin, collected by centrifugation, washed twice with PBS and stained with 5 μL of annexin V in 250 μL binding buffer for 10 min at room temperature. Cells were again washed with PBS, re-suspended in 250 μL binding buffer and stained with 10 μL of PI. Apoptotic cells were determined using the FACSFortessa (BD BioSciences, CA) [33].

### Cell cycle analysis

Cell cycle analysis was performed by PI staining for DNA content and flow cytometric analysis. Briefly, after treatment, adherent cells were collected using trypsin-EDTA by centrifugation at 10000 rpm for 5 min, and washed twice with ice cold PBS. During the last spin, 5ul PI was added for every ml of hypotonic buffer (0.1% Sodium Citrate, 0.1% Triton X-100), and incubated on ice in the dark (at least 20 min). Stained cells were analyzed (at least 20,000 events per sample) with a FACSFortessa flow cytometer (BD BioSciences, CA).

### Statistical analysis

Results were shown as means ± standard deviations of three independent experiments. The difference between two groups was analyzed using Student’s t-test, and a *P*-value < 0.05 was considered statistically significant.

## Results

### Determination of BRCA1 expression and HR functionality in ovarian cancer (OC) cell lines

**Fig. 1:**
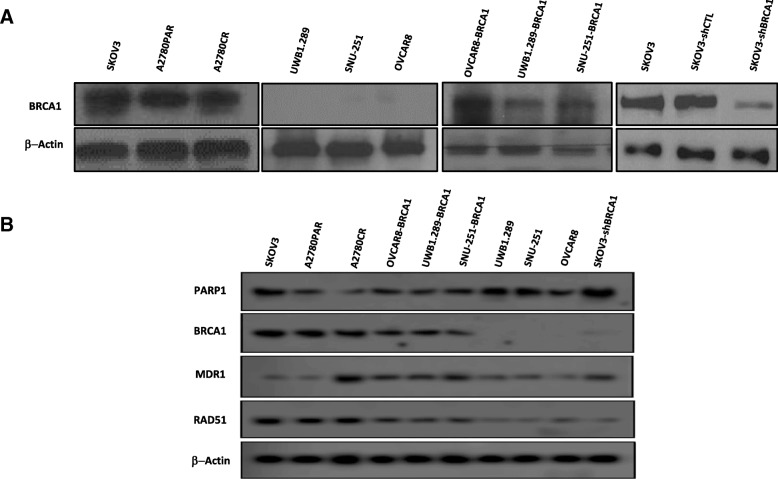
Expression of BRCA1 and HR-related genes in ovarian cancer lines. **a** BRCA1 protein expression in SKOV3, A2780PAR, A2780CR, UWB1.289, Snu-251, OVCAR8, OVCAR8-BRCA1, UWB1.289-BRCA1, SNU-251-BRCA1, SKOV3, SKOV3-shCTL and SKOV3-shBRCA1 cell lines. **b** Protein expression of DNA-damage repair and multi-resistance drug pathway genes, PARP1, RAD51 and MDR1, including BRCA1 in OC cell lines

BRCA1 protein expression was observed in SKOV3, A2780PAR and A2780CR cells as compared to UWB1.289, SNU-251 and OVCAR8 cells showed no expression. Furthermore, stable SKOV3-shBRCA1 knockdown was developed, and 70–80% down regulation of BRCA was observed (Fig. 1a). In addition, after stable restoration of BRCA1 in mutated and methylated cells BRCA1 protein expression was increased (Fig. 1a).

Moreover, we have evaluated the PARP1 protein expression levels in these OC cell lines and we have found different expression levels in the cell lines. Interestingly chemo-treated A2780CR as compared to chemo-naive A2780PAR have showed low expression of PARP1 (Fig. 1b), similarly as described by our group [28].

Furthermore, BRCA1-mutated and methylated cells expressed reduced levels of RAD51 as compared with BRCA1 wild-type, suggesting a deficiency in HR function and also corroborated with previous results [34]. Finally, multidrug resistance protein (MDR1), involved in drug resistance, was also evaluated. The development of MDR1 could lead to a treatment failure in patients with ovarian cancer, and this severely limits the ultimate success of chemotherapy in the clinic in patients [35]. We have found that chemo naive cells have showed low levels of MDR1 as compared to chemo-treated (Fig. 1b).

### HR deficiency sensitizes ovarian cancer cells to PARP inhibitors

**Fig. 2:**
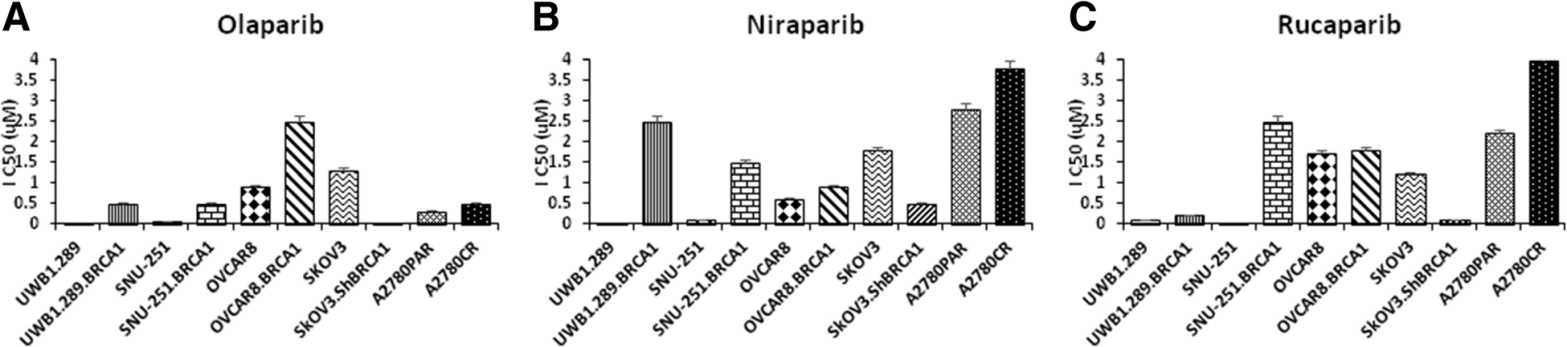
Ovarian cancer cells sensitivity to different PARP inhibitors. The half maximal inhibitory concentration (IC50) of different OC cells after treatment with (**a**) olaparib, (**b**) niraparib and (**c**) rucaparib, assessed by clonogenic assays. Data represents the mean ± SEM of triplicates of three independent experiments

Cell lines with different HR status were exposed to different doses of PARPi (0.5 μM to 4 μM). OC cells with BRCA1-mutated and HR-deficient genes display higher sensitivity to PARPi compared to cells with wild-type, methylated or restored BRCA1 (Fig. 2). In contrast, OC cells that harbour a functional HR needed to be expossed to higher doses of olaparib (Fig. 2a), rucaparib (Fig. 2b) and niraparib (Fig. 2c) to achieve growth inhibition (Additional file 1: Table S1). Altogether, these results confirmed that OC cells with mutated BRCA1 and deficient HR-related genes are more sensitive to PARPi similarly to other studies [36, 37].

Currently, olaparib is used as a maintenance therapy in patients after response to cisplatin therapy [38]. We evaluated whether olaparib prior to chemotherapy might be more effective treatment. SKOV3, SKOV3-shBRCA1, SNU-251 and SNU-251-BRCA1 cells were plated for clonogenic assays and cell colonies were counted to determine cell growth. Cells were treated with increasing concentrations of olaparib follow by cisplatin (2μg) or increasing dose of cisplatin follows by olaparib (0.1 μM and 0.5 μM).

**Fig. 3:**
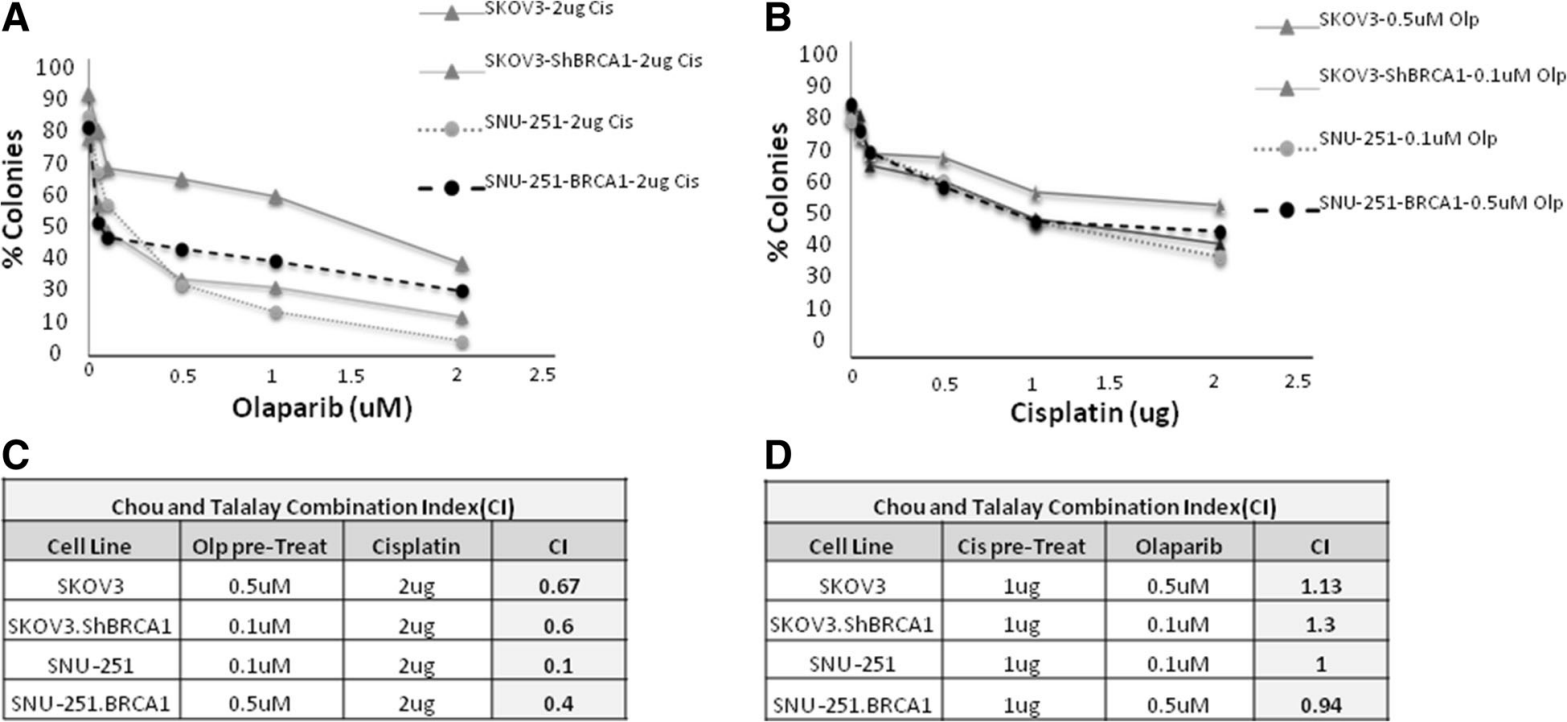
Ovarian cancer cells pre-treated with PARPi followed by cisplatin showed strong synergism. **a** SKOV3, SKOV3-shBRCA1 and SNU-251, SNU-251-BRCA1 cells were pre-treated with increases doses of olaparib (0-2uM) follow by 2 μg of cisplatin for 7 days and survival was determined using the clonogenic assay. **b** Cells were pre-treated with increases doses of cisplatin (0-2 μg) follow by olaparib (0.1uM-0.5uM) for 7 days and their effect on cell survival was evaluated using the clonogenic assay. The evaluation of combination index (CI) for pretreated olaparib (**c**) and cisplatin pretreated (**d**) in OC cells was calculated where CI < 1 indicates synergy between the two drugs and CI > 1 indicates an additive effect. Results are presented as means ± SEM for triplicates of three independent experiments

Cells pre-treated with olaparib or rucaparib followed by cisplatin had significantly decreased cell growth compared to the inverse sequence (Fig. 3a and b, Additional file 2: Figure S2). To further determine the nature of the interaction we used the multiple drug effects analysis method by Chou and Talalay. In both cell lines the combination of olaparib followed cisplatin was synergistic, with a CI between 0.1–0.6. In contrast, cells pre-treated with cisplatin followed by olaparib display additive effect (CI > 1) (Fig. 3c, d). Similar results were observed in UWB1.289, UWB1.289-BRCA1, OVCAR8, OVCAR8-BRCA1, A2780PAR and A2780CR (Additional file 3: Figure S1).

### Olaparib pre-treatment followed by cisplatin induce cell death and cell cycle arrest

**Fig. 4:**
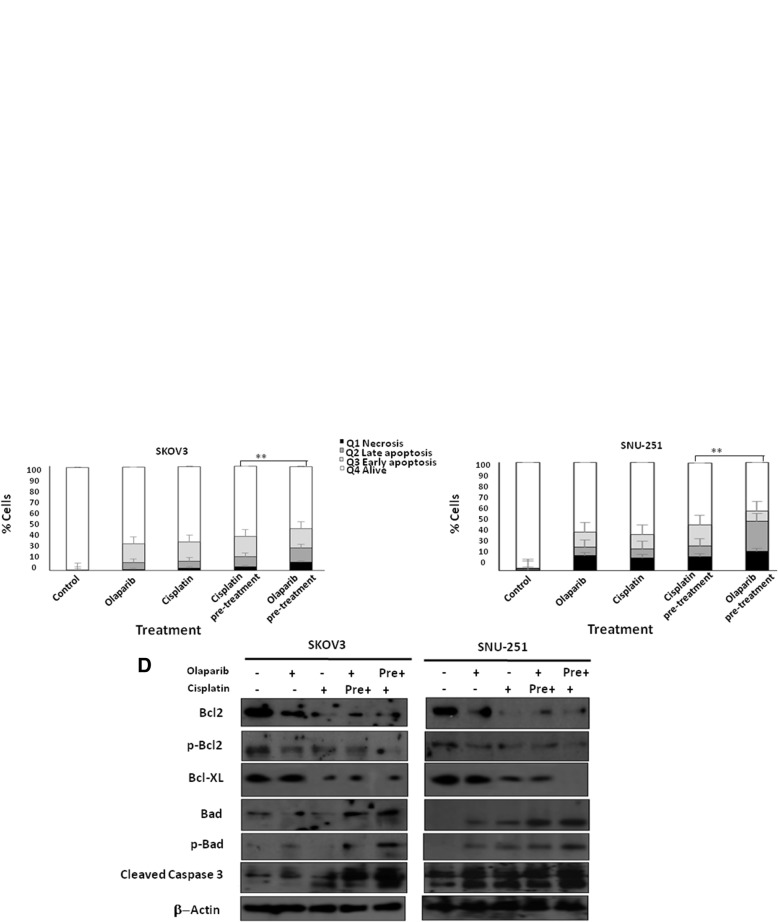
Pre-treatment with PARPi followed by cisplatin increases cell death. SKOV3 (**a**) and (**b**) and SNU-251 (**a**) and (**c**) cells were treated with different combinations of olaparib and cisplatin for 48 h, then apoptotic rates were assessed using Annexin V/PI double staining followed by flow cytometry analysis. **d** Protein expression of pro and anti-apoptotic proteins (Bcl2, p-Bcl, Bcl-XL, Bad, p-Bad, cleaved caspase3) were examined by western blot in SKOV3 and SNU-251 cells. Results are presented as means ± SEM for triplicates of three independent experiments,*p value < 0.05

We further investigated the effect of these sequence treatments on regulating apoptosis and cell cycle. First, we studied the effects of these treatments by quantifying the apoptotic cells using Annexin V/PI double staining assay (Fig. 4a). As presented in Fig. 4a, b and c, we found that olaparib or cisplatin monotherapy induced cell death in ~ 23–30% of SKOV3 cells, and ~ 30–40% of SNU-251 cells in comparison with control cells. Pre-treatment with cisplatin followed by olaparib induced cell death up to ~ 35% in SKOV3 and ~ 42% in SNU-251 cells. On the other hand, pre-treatment with olaparib followed by cisplatin increased cell death to ~ 52% (*p* = 0.02) of SKOV3 cells and ~ 70% (*p* = 0.002) of SNU-251 cells (Fig. 4b and c). Moreover, these results were similar in A2780 PAR, A2780CR, OVCAR8 and UWB1.289 cell lines (data not shown). Furthermore, we evaluated the pro-survival proteins Bcl2, p-Bcl2 and Bcl-XL. Our results showed that these proteins were all significantly down regulated in SKOV3 and SNU-251 cells, while pro-apoptotic proteins Bad, p-Bad and cleaved caspase-3 were up regulated, and this was more pronounced in cells after pre-treatment with olaparib (Fig. 4d).

**Fig. 5:**
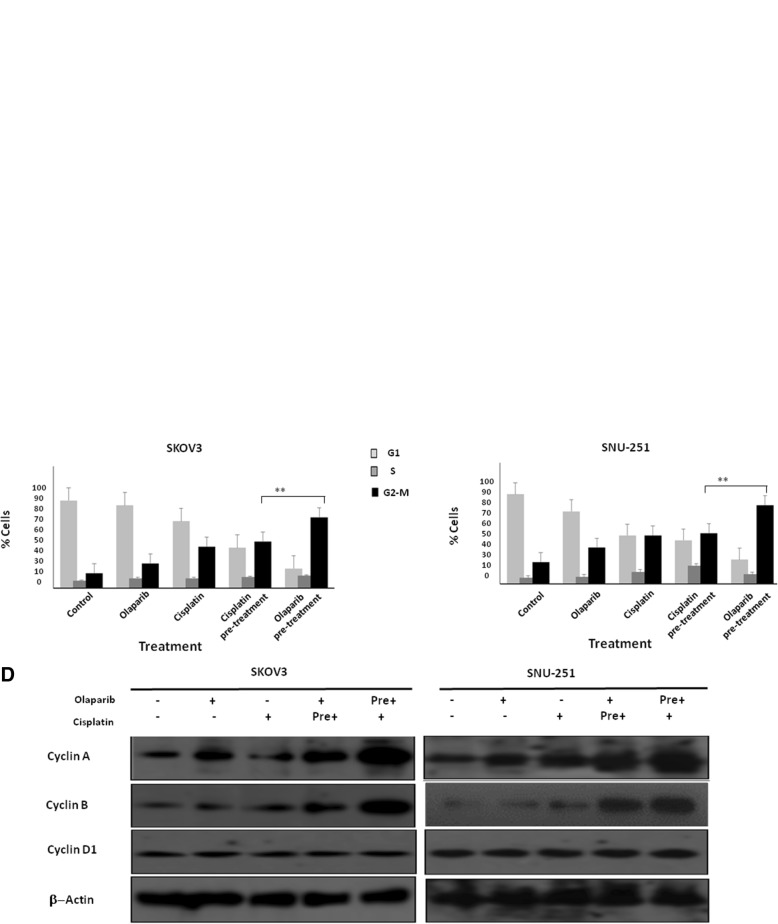
Cell cycle analysis. SKOV3 (**a** and **b**) and SNU-251(**a** and **c**) cells were treated with different combinations of olaparib and cisplatin for 48 h, cells were synchronized and cell cycle analysis were performed using flow cytometry. **d** Protein expression of cell cycle related proteins (cyclin A, cyclin B and cyclin D1) were examined by western blot. Results are presented as means ± SEM for triplicates of three independent experiments, *p value < 0.05

Next, we analyzed the effect of these treatment combinations on cell cycle progression. Olaparib pre-treatment followed by cisplatin resulted in G2/M arrest in up to ~ 60% (*p* < 0.01) of SKOV3 and ~ 73% (*p* < 0.001) of SNU-251 cells (Fig. 5a-c) compared to ~ 25–40% using the inverse sequence. Moreover, the arrest at this phase was also confirmed by evaluating cyclins (Fig. 5d). While we did not observe any change in cyclin D1, a significant increase in cyclin A and cyclin B was observed after olaparib pre-treatment in SKOV3 and SNU-251 cells, consistent with the G2/M arrest observed in the cell cycle analysis. Taken together these results highlight the increased inhibitory effect of olaparib pre-treatment followed by Cisplatin in cell proliferation and cell growth, correlated to cell cycle arrest and induction of apoptosis.

## Discussion

PARPi is currently indicated as maintenance treatment for recurrent HGSOC following second line platinum-based therapy [39]. Previously, we reported that PARP1 protein levels were reduced following chemotherapy in vitro and in vivo [28]. These findings triggered the rationale of our study, raising the question whether PARPi might be more efficient prior to chemotherapy, compared to the present regimen that have focused on tumors previously exposed to standard chemotherapy [40–42].

Recent reports showed that by trapping PARP1 on DNA via PARPi is more effective in slowing cell proliferation than knocking down PARP1 [43]. We found that tumors harbouring undetectable levels of PARP1 protein, still transcribe PARP1 mRNA, suggesting that the down regulation of PARP1 protein by chemotherapy is likely post-translational. So, this led to the strategy of giving PARPi prior to chemotherapy to get the maximum efficacy. This work describes for the first time the improved results of using PARPi prior to chemotherapy in vitro.

Here, we have shown that OC cell lines with different HR status and MDR1 levels, especially A2780PAR and A2780CR, responded better after olaparib pre-treatment, suggesting that PARPi can maximize the benefit of chemotherapy and delay the process of chemo-resistance in the OC cell lines. Pre-treatment further favored the ratio of pro-apoptotic Bcl-2 family members (Bax, Bad) to anti-apoptotic Bcl-2 family (Bcl-2 and Bcl-XL) members with increased cell apoptosis as indicated by flow cytometry analysis and western blot. Interestingly, we have found that the sequential treatment with olaparib followed by cisplatin synergistically inhibited HGSOC cell proliferation at clinically achievable concentrations.

Moreover, is important to mention the limitations of our study. The use of commercial cell lines differ from patient tumors which are often more heterogeneous. BRCA1 mutation confer a genetic susceptibility to ovarian cancer by almost 60%, however in further studies will be interesting to evaluate the influence of BRCA2 mutation. In addition, studies including xenograft models are needed.

## Conclusion

This study is the first which indicated that pre-treatment with PARPi followed chemotherapy in vitro is more efficient in growth inhibition and induction of cell apoptosis as compared to the administration of standard chemotherapy followed by PARPi. Furthermore, as a combined effect of PARPi follow chemotherapy has greater synergism in BRCA1 silenced cells and to a lesser degree in BRCA1 wild-type cells, which provide a therapeutic option for HGSOC patients independent of their BRCA1 status. Moreover, we have indicated a new direction of employing PARPi as a first line of treatment, suggesting that pre-treatment with PARPi follow chemotherapy as frontline therapy might yield significantly better outcome in HGSOC, and could also be explored in other patients with homologous recombination-deficient cancers. Altogether suggest a new treatment strategy that needs to be further evaluated in comparison with the currently available standard treatment.

## Additional files

Additional file 1: **Figure S1.** Ovarian cancer cells pre-treated with PARPi followed by cisplatin showed strong synergism. UWB1.289, UWB1.289-BRCA1, OVCAR8, OVCAR8-BRCA1 cells (A) A2780PAR and A2780CR cells (E) were pre-treated with increases doses of olaparib (0-2 uM) follow by 2 µg of cisplatin for 7 days and their effect on cell survival was evaluated using the clonogenic assays. (B and F) Cells were pre-treated with increases doses of cisplatin (0-2 µg) follow by olaparib (0.1 uM and 0.5 uM) for 7 days and their effect on cell survival was evaluated using the clonogenic assay. The evaluation of combination index (CI) for pretreated olaparib (C and G) and cisplatin pretreated (D and H) in OC cells was calculated where CI<1 indicates synergy between the two drugs and CI>1 indicates an additive effect. Results are presented as means ± SEM for triplicates of three independent experiments. (PPTX 34 kb)

Additional file 2: **Figure S2.** Ovarian cancer cells pre-treated with PARPi followed by cisplatin showed strong synergism. (A) SKOV3, SKOV3-shBRCA1 and SNU-251, SNU-251-BRCA1 cells were pre-treated with increases doses of rucaparib (0-2 uM) follow by 2 µg of Cisplatin for 7 days and cell survival was determined using the clonogenic assays. (B) Cells were pre-treated with increases doses of cisplatin (0-2 µg) follow by rucaparib (0.1 uM -0.5 uM) for 7 days and their effect on cell survival was evaluated using the clonogenic assays. Results are presented as means ± SEM for triplicates of three independent experiments. (PPTX 82 kb)

Additional file 3: **Table S1.** IC50 concentration of PARPi in different OC cell lines. The table depicts a summary of the median inhibitory concentrations (IC50) of olaparib, rucaparib, niraparib and cisplatin in different OC cell lines assessed by clonogenic assay. (PPTX 842 kb)

## Abbreviations

*BRCA1/2*: Breast susceptible cancer gene
CI: Combination Index
DSB: Double strand break
HGSOC: High Grade Serous Ovarian Cancer
HR: Homologous recombination
IC50: Inhibitory concentration
PARP: Poly (ADP-ribose) polymerase
SSB: Single strand break
