# Supplementary material for: Sequential therapeutic targeting of ovarian Cancer harboring dysfunctional BRCA1
Source: BMC Cancer. 2019 Jan 10;19:44. doi: 10.1186/s12885-018-5250-4 (PMC6327434; doi:10.1186/s12885-018-5250-4)
Supplement: Supplementary file 1 — Figure S1. Ovarian cancer cells pre-treated with PARPi followed by cisplatin showed strong synergism. UWB1.289, UWB1.289-BRCA1, OVCAR8, OVCAR8-BRCA1 cells (A) A2780PAR and A2780CR cells (E) were pre-treated with increases doses of olaparib (0-2 uM) follow by 2 µg of cisplatin for 7 days and their effect on cell survival was evaluated using the clonogenic assays. (B and F) Cells were pre-treated with increases doses of cisplatin (0-2 µg) follow by olaparib (0.1 uM and 0.5 uM) for 7 days and their effect on cell survival was evaluated using the clonogenic assay. The evaluation of combination index (CI) for pretreated olaparib (C and G) and cisplatin pretreated (D and H) in OC cells was calculated where CI<1 indicates synergy between the two drugs and CI>1 indicates an additive effect. Results are presented as means ± SEM for triplicates of three independent experiments. (PPTX 34 kb) [file 12885_2018_5250_MOESM1_ESM.pptx]

## Slide 1
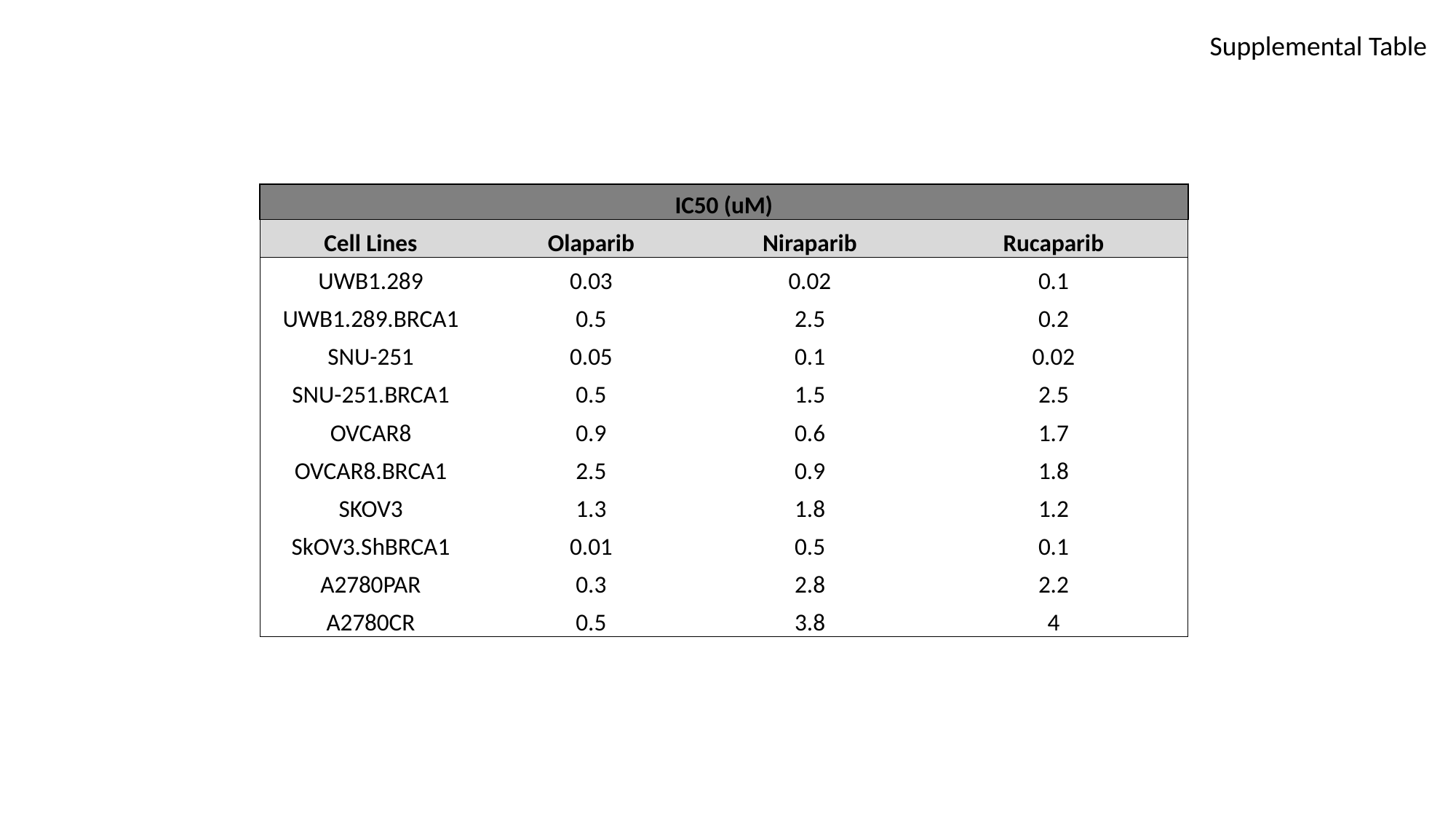

Supplemental Table
| IC50 (uM) | | | |
| --- | --- | --- | --- |
| Cell Lines | Olaparib | Niraparib | Rucaparib |
| UWB1.289 | 0.03 | 0.02 | 0.1 |
| UWB1.289.BRCA1 | 0.5 | 2.5 | 0.2 |
| SNU-251 | 0.05 | 0.1 | 0.02 |
| SNU-251.BRCA1 | 0.5 | 1.5 | 2.5 |
| OVCAR8 | 0.9 | 0.6 | 1.7 |
| OVCAR8.BRCA1 | 2.5 | 0.9 | 1.8 |
| SKOV3 | 1.3 | 1.8 | 1.2 |
| SkOV3.ShBRCA1 | 0.01 | 0.5 | 0.1 |
| A2780PAR | 0.3 | 2.8 | 2.2 |
| A2780CR | 0.5 | 3.8 | 4 |
