# Supplementary material for: Sequential therapeutic targeting of ovarian Cancer harboring dysfunctional BRCA1
Source: BMC Cancer. 2019 Jan 10;19:44. doi: 10.1186/s12885-018-5250-4 (PMC6327434; doi:10.1186/s12885-018-5250-4)
Supplement: Supplementary file 2 — Figure S2. Ovarian cancer cells pre-treated with PARPi followed by cisplatin showed strong synergism. (A) SKOV3, SKOV3-shBRCA1 and SNU-251, SNU-251-BRCA1 cells were pre-treated with increases doses of rucaparib (0-2 uM) follow by 2 µg of Cisplatin for 7 days and cell survival was determined using the clonogenic assays. (B) Cells were pre-treated with increases doses of cisplatin (0-2 µg) follow by rucaparib (0.1 uM -0.5 uM) for 7 days and their effect on cell survival was evaluated using the clonogenic assays. Results are presented as means ± SEM for triplicates of three independent experiments. (PPTX 82 kb) [file 12885_2018_5250_MOESM2_ESM.pptx]

## Slide 1
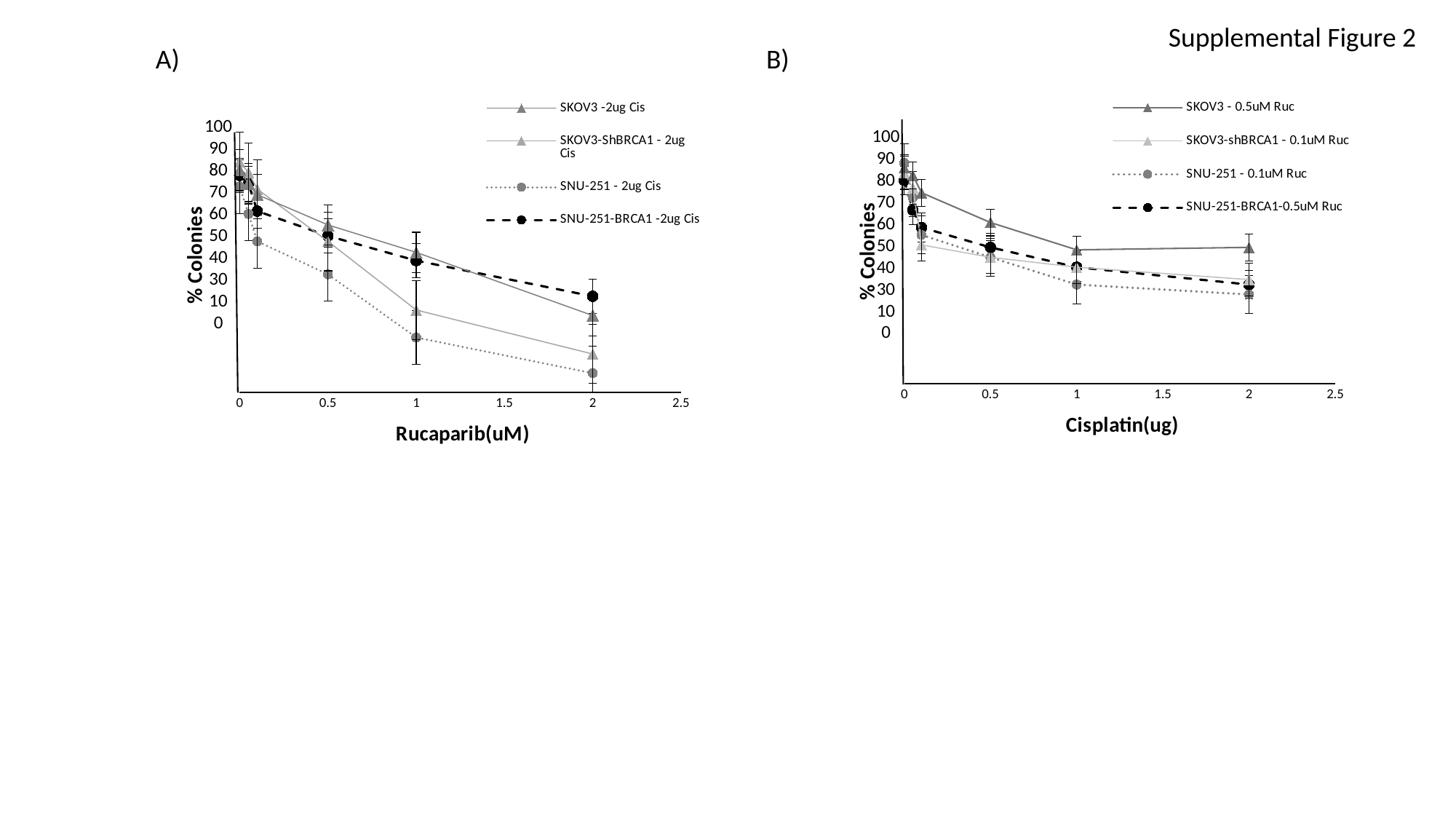

Supplemental Figure 2
A)
B)
### Chart
| Category | SKOV3 - 0.5uM Ruc | SKOV3-shBRCA1 - 0.1uM Ruc | SNU-251 - 0.1uM Ruc | SNU-251-BRCA1-0.5uM Ruc |
|---|---|---|---|---|100
90
80
70
60
50
40
30
10
0
### Chart
| Category | SKOV3 -2ug Cis | SKOV3-ShBRCA1 - 2ug Cis | SNU-251 - 2ug Cis | SNU-251-BRCA1 -2ug Cis |
|---|---|---|---|---|100
90
80
70
60
50
40
30
10
0
