# Supplementary material for: Sequential therapeutic targeting of ovarian Cancer harboring dysfunctional BRCA1
Source: BMC Cancer. 2019 Jan 10;19:44. doi: 10.1186/s12885-018-5250-4 (PMC6327434; doi:10.1186/s12885-018-5250-4)
Supplement: Supplementary file 3 — Table S1. IC50 concentration of PARPi in different OC cell lines. The table depicts a summary of the median inhibitory concentrations (IC50) of olaparib, rucaparib, niraparib and cisplatin in different OC cell lines assessed by clonogenic assay. (PPTX 842 kb) [file 12885_2018_5250_MOESM3_ESM.pptx]

## Slide 1
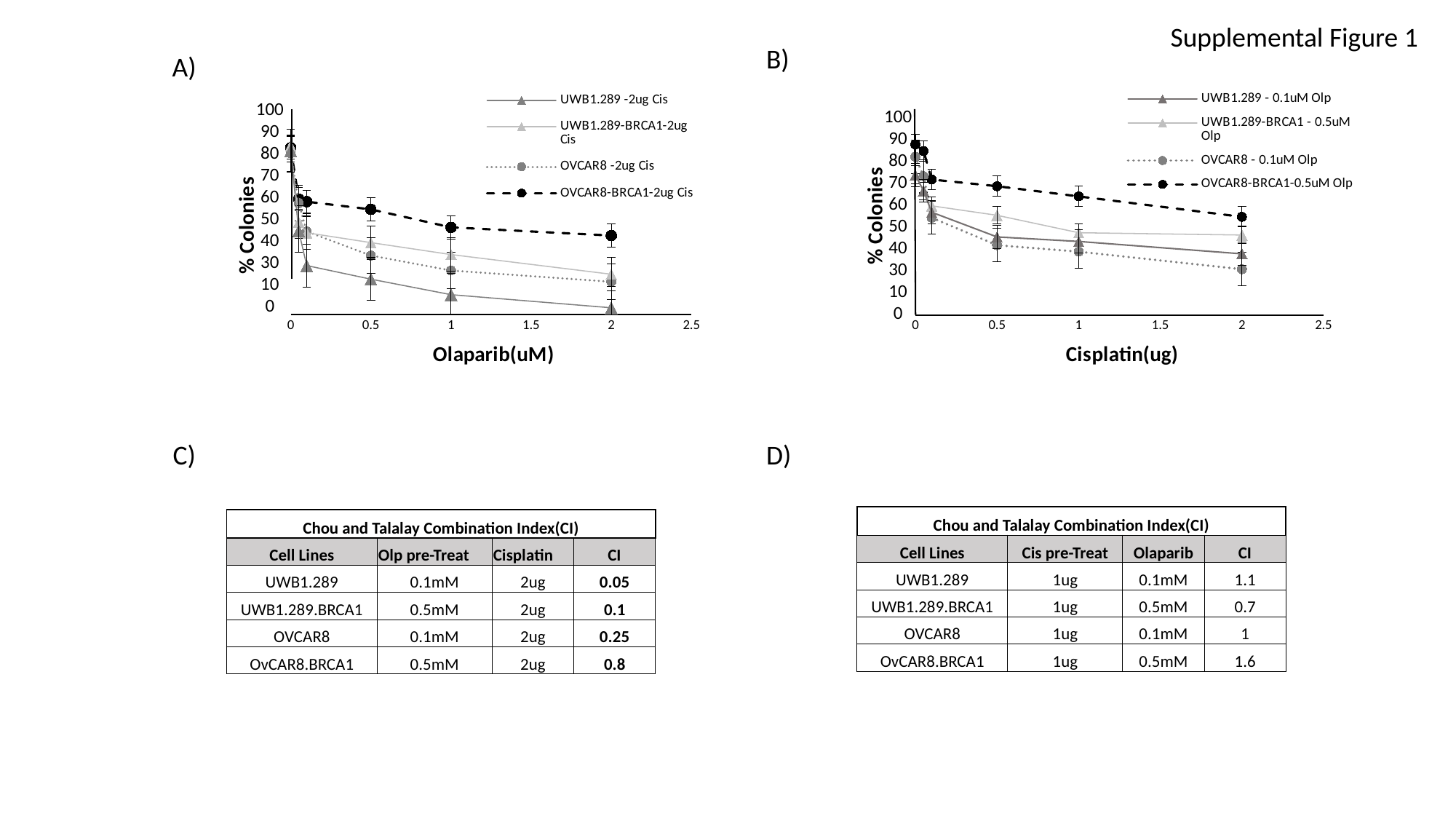

Supplemental Figure 1
B)
A)
### Chart
| Category | UWB1.289 -2ug Cis | UWB1.289-BRCA1-2ug Cis | OVCAR8 -2ug Cis | OVCAR8-BRCA1-2ug Cis |
|---|---|---|---|---|
### Chart
| Category | UWB1.289 - 0.1uM Olp | UWB1.289-BRCA1 - 0.5uM Olp | OVCAR8 - 0.1uM Olp | OVCAR8-BRCA1-0.5uM Olp |
|---|---|---|---|---|100
90
80
70
60
50
40
30
10
0
100
90
80
70
60
50
40
30
10
0
C)
D)
| Chou and Talalay Combination Index(CI) | | | |
| --- | --- | --- | --- |
| Cell Lines | Cis pre-Treat | Olaparib | CI |
| UWB1.289 | 1ug | 0.1mM | 1.1 |
| UWB1.289.BRCA1 | 1ug | 0.5mM | 0.7 |
| OVCAR8 | 1ug | 0.1mM | 1 |
| OvCAR8.BRCA1 | 1ug | 0.5mM | 1.6 |
| Chou and Talalay Combination Index(CI) | | | |
| --- | --- | --- | --- |
| Cell Lines | Olp pre-Treat | Cisplatin | CI |
| UWB1.289 | 0.1mM | 2ug | 0.05 |
| UWB1.289.BRCA1 | 0.5mM | 2ug | 0.1 |
| OVCAR8 | 0.1mM | 2ug | 0.25 |
| OvCAR8.BRCA1 | 0.5mM | 2ug | 0.8 |

## Slide 2
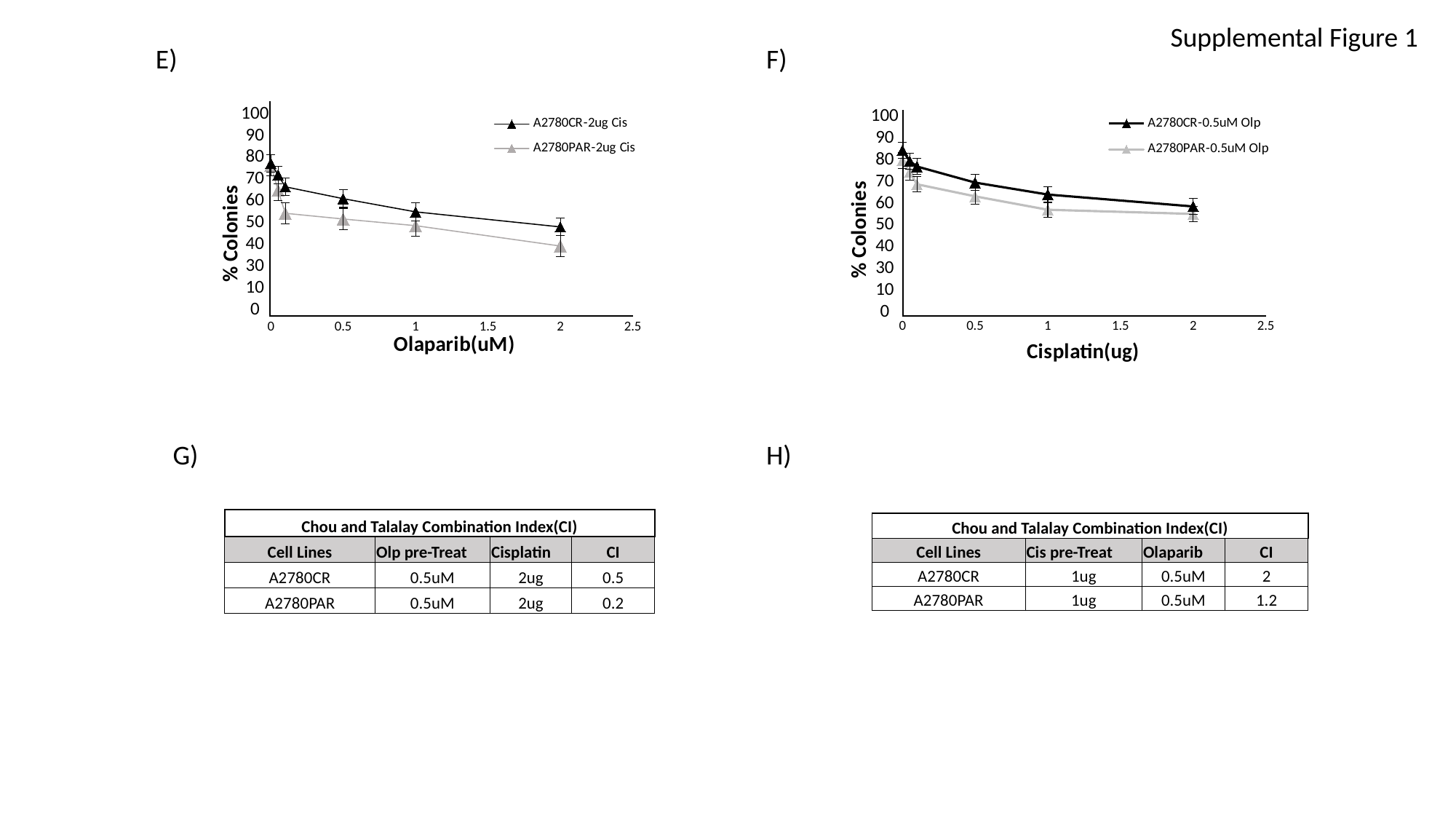

Supplemental Figure 1
E)
F)
### Chart
| Category | A2780CR-2ug Cis | A2780PAR-2ug Cis |
|---|---|---|100
90
80
70
60
50
40
30
10
0
### Chart
| Category | A2780CR-0.5uM Olp | A2780PAR-0.5uM Olp |
|---|---|---|100
90
80
70
60
50
40
30
10
0
G)
H)
| Chou and Talalay Combination Index(CI) | | | |
| --- | --- | --- | --- |
| Cell Lines | Olp pre-Treat | Cisplatin | CI |
| A2780CR | 0.5uM | 2ug | 0.5 |
| A2780PAR | 0.5uM | 2ug | 0.2 |
| Chou and Talalay Combination Index(CI) | | | |
| --- | --- | --- | --- |
| Cell Lines | Cis pre-Treat | Olaparib | CI |
| A2780CR | 1ug | 0.5uM | 2 |
| A2780PAR | 1ug | 0.5uM | 1.2 |
